# Application of a New Genetic Deafness Microarray for Detecting Mutations in the Deaf in China

**DOI:** 10.1371/journal.pone.0151909

**Published:** 2016-03-28

**Authors:** Hong Wu, Yong Feng, Lu Jiang, Qian Pan, Yalan Liu, Chang Liu, Chufeng He, Hongsheng Chen, Xueming Liu, Chang Hu, Yiqiao Hu, Lingyun Mei

**Affiliations:** 1 ENT Department, Xiangya Hospital, Central South University, Changsha, Hunan, China; 2 National Laboratory of Medical Genetics of China, School of Life Science, Central South University, Changsha, Hunan, China; 3 Province Key Laboratory of Otolaryngology Critical Diseases, Xiangya Hospital, Central South University, Changsha, Hunan, China; 4 ENT Department, Changsha First Hospital, Changsha, Hunan, China; NIDCR/NIH, UNITED STATES

## Abstract

**Objective:**

The aim of this study was to evaluate the GoldenGate microarray as a diagnostic tool and to elucidate the contribution of the genes on this array to the development of both nonsyndromic and syndromic sensorineural hearing loss in China.

**Methods:**

We developed a microarray to detect 240 mutations underlying syndromic and nonsyndromic sensorineural hearing loss. The microarray was then used for analysis of 382 patients with nonsyndromic sensorineural hearing loss (including 15 patients with enlarged vestibular aqueduct syndrome), 21 patients with Waardenburg syndrome, and 60 unrelated controls. Subsequently, we analyzed the sensitivity, specificity, and reproducibility of this new approach after Sanger sequencing-based verification, and also determined the contribution of the genes on this array to the development of distinct hearing disorders.

**Results:**

The sensitivity and specificity of the microarray chip were 98.73% and 98.34%, respectively. Genetic defects were identified in 61.26% of the patients with nonsyndromic sensorineural hearing loss, and 9 causative genes were identified. The molecular etiology was confirmed in 19.05% and 46.67% of the patients with Waardenburg syndrome and enlarged vestibular aqueduct syndrome, respectively.

**Conclusion:**

Our new mutation-based microarray comprises an accurate and comprehensive genetic tool for the detection of sensorineural hearing loss. This microarray-based detection method could serve as a first-pass screening (before next-generation-sequencing screening) for deafness-causing mutations in China.

## Introduction

Hearing loss is the most common sensory-nerve disease worldwide [1], with approximately 250 million people suffering from moderate to severe hearing loss [2]. Moreover, one child in 1,000 is estimated to be born with prelingual hearing loss [3]. The 2006 National Sample Survey in China [4] revealed that hearing impairment affected roughly 20 million people, accounting for 24.16% of all people with disabilities. Furthermore, approximately 30,000 babies are born with congenital hearing impairment annually in China [4], and nearly half of the cases of congenital deafness are estimated to be associated with genetic factors [5]. Genetics studies have shown that 77% of hereditary deafness is autosomal recessive, while 22% is autosomal dominant, 1% is associated with X chromosomal mutations, and <1% is associated with maternally inherited mitochondrial genes.

In hereditary hearing impairment, roughly 70% of the cases are nonsyndromic [6] and, as of August 2015, >80 pathogenic nuclear genes associated with this disorder have been cloned. Moreover, 2 mitochondrial genes were found to be associated with nonsyndromic sensorineural hearing loss (SNHL) (http://hereditaryhearingloss.org/). In particular, *GJB2* (OMIM: 121011), *GJB3* (OMIM: 603324), *SLC26A4* (OMIM: 605646), and the mitochondrial gene *MT-RNR1* (OMIM: 561000) are the 4 major causative genes of hereditary nonsyndromic SNHL in China [7]. The incidence of positive genetic errors in these 4 genes was reported to be 43.58% [6]. However, beyond the mutations in these 4 genes, it is unclear which genetic defects are the next most prevalent among SNHL patients in China. To address this gap in our knowledge and to develop a microarray that is more comprehensive than the ones used to date, we selected key mutations from a larger number of deafness genes than were previously analyzed; these mutations were selected based on the prevalence at which they are reported in the literature.

Currently, >400 types of syndromes are recognized to be associated with hearing impairment, accounting for 30% of prelingual deafness [6]. The most common autosomal-recessive type of syndromic deafness is Usher syndrome (US; OMIM: 276900). While US is known to affect 50% of the deaf-blind population in the USA [8], few studies of this disease have been conducted in China. Pendred syndrome (PS; OMIM: 274600) is the second most common type of autosomal-recessive syndromic deafness and is characterized by severe to profound sensorineural deafness and goiter [9]. Meanwhile, the most common autosomal-dominant syndromic deafness is Waardenburg syndrome (WS), which is mainly characterized by impaired hearing and pigmentation abnormalities and is highly heterogeneous both clinically and genetically, with WS1 (OMIM: 193500) and WS2 (OMIM: 193510) being the most prevalent forms of WS in China [10]. The microarray chip we developed features gene mutations related to each of the aforementioned common types of syndromic SNHL for the following reasons: First, the incidences of these 3 syndromes are high; second, the 3 syndromes can directly and markedly affect quality of life; third, because of their phenotypic and genotypic heterogeneity, the 3 syndromes might be underdiagnosed or highly challenging to detect. Genetic screening is a currently available method for early diagnosis, which helps to prevent or predict disease and thus considerably alleviate the disease burden on patients and their families. Therefore, we considered it critical to include mutations related to these 3 deafness syndromes in our microchip assay.

Recent developments in molecular genetics have led to the design of diverse gene diagnostic tools. However, there is currently no detection method that covers both syndromic and nonsyndromic hearing loss. Moreover, a comprehensive genetic array for the Chinese SNHL population has yet to be developed. This study was therefore conducted, not only to evaluate the GoldenGate array as a diagnostic tool, but also to further elucidate the contribution of the genes on this array to the development of both nonsyndromic and syndromic SNHL in China.

## Materials and Methods

### Samples

A total of 403 unrelated Han Chinese patients with SNHL were recruited between 2014 and 2015 from the Otolaryngology Department of Xiangya Hospital, Central South University. We recruited patients in whom deafness resulted from unclear causative factors, but excluded patients in whom clear causative factors were identified such as noise exposure, trauma (exception for the patients who are identified with enlarged vestibular aqueduct by medical imaging), intrauterine infection, poisoning, or tumors. All participants were probands and were divided into 382 cases of nonsyndromic SNHL (including 15 of enlarged vestibular aqueduct (EVA) syndrome) and 21 cases of WS based on clinical history, physical examination, audiological examination, and imaging tests. All patients presented with moderate to profound bilateral SNHL in pure-tone audiometry and/or auditory brainstem response testing. Hearing loss was classified into mild (26–40 dB), moderate (41–55 dB), moderate severe (56–70 dB), severe (71–90 dB), and profound (>90 dB). Genomic DNA was isolated from the blood of each patient using the phenol/chloroform extraction method. As a control for detection, we used DNA samples from 60 healthy unrelated people from the Chinese population who did not show any sign of hearing loss.

This study was approved by the Xiangya Ethics Committee, and written informed consent was obtained from all participants or their parents (when participants were under 18 years old).

### Gene selection and array design

We comprehensively searched the literature available on PubMed, Embase, the Chinese National Knowledge Infrastructure (CNKI), and the Chinese Wanfang Literature Database in order to estimate the likely frequency of mutations in causative genes for SNHL in the Chinese population, and then selected an initial set of genes that were previously implicated in SNHL for inclusion. The paramount criterion for inclusion was that the mutations had to have been reported >2 times, and primacy was given to the mutations that had been reported in the Chinese population. In the end, the GoldenGate custom microarray (Illumina, San Diego, CA, USA) included 240 mutations selected from 46 nuclear genes (such as *GJB2*, *GJB6*, *GJB3*, and *SLC26A4*) and one mitochondrial gene, *MT-RNR1*; a detailed list of the variants is provided in S1 Table. The mutation list included single-nucleotide changes as well as deletions. For each of the 240 mutations on the microarray, 60-bp oligonucleotides were constructed in both directions based on the wild-type sequence of each gene.

The 240 mutations covered all currently characterized mutations involved in the etiology of either nonsyndromic or syndromic SNHL, and were comprised of (1) mutations covering the mitochondrial gene and the 46 autosomal deafness genes; (2) 200 nonsyndromic SNHL variants (43 dominant inherited variants, 152 recessive inherited variants, 1 maternally inherited variant, and 4 X-linked variants) and 40 common syndromic SNHL variants related to WS, PS, and US (Table 1); and (3) 8 of the most common mutation hotspots in the Chinese population: *GJB2* c.35delG, *GJB2* c.235delC, *GJB2* c.299-300delAT, *GJB3* c.538C>T, *GJB3* c.1174A>T, *SLC26A4* c.IVS7-2A>G, *SLC26A4* c.2168 A>G, and mitochondrial *12rsRNA* 1555A>G.

**Table 1:** Distribution of the detection sites on the microarray.

	Category	Number of mutations	Number of genes
Nonsyndromic hearing loss	Recessive inheritance	152	30
	Dominant inheritance	43	13
	X-linked inheritance	4	1
	Maternal inheritance	1	1
Syndromic hearing loss	Waardenburg Syndrome	14	5
	Pendred Syndrome	8	2
	Usher Syndrome	18	2

### GoldenGate genotyping assay

In the GoldenGate genotyping assay (Illumina) protocol, allele-specific extension methods are used and PCR-amplification reactions are conducted at high multiplex levels. The entire process is completed in 3 days, as described below.

**Day 1.** Preparation, precipitation, and resuspension of samples in single-use DNA (SUD) plates

DNA samples were normalized to a concentration of 50 ng/μL with a Tris-EDTA buffer (10 mM Tris-HCl [pH 8.0], 1 mM EDTA), after which 5 μL of MS1 reagent and 5 μL of the normalized DNA sample were added to each well of the SUD plates; the plates were then incubated at 95°C for exactly 30 min. Next, 5 μL of PS1 and 15 μL of 2-propanol were added to the wells of the SUD plates to precipitate the DNA, and excess MS1 DNA-activation reagent was removed. Lastly, 10 μL of RS1 was added to each well to resuspend the DNA.

Preparation of the allele-specific extension (ASE) plates

Ten microliters of OPA, 30 μL of OB1, and 10 μL of the biotinylated sample from the SUD plates were added to ASE plates. Plates were then sealed and placed on a 70°C heat block, after which the temperature was immediately reset to 30°C. The ASE plates were incubated on the heat block for 16 h and the query oligos for each target sequence of interest were allowed to anneal to the biotinylated genomic-DNA samples.

**Day 2.** Addition of the master mix for extension and ligation (MEL).

To wash off nonspecifically hybridized and excess oligos, 50 μL of AM1 and 50 μL of UB1 were added to the ASE plates. After washing, 37 μL of MEL was added to each DNA sample, and the extension and ligation reaction was allowed to proceed at 45°C for exactly 15 min.

PCR amplification

Sixty-four microliters of Titanium Taq DNA Polymerase, 50 μL of UB1, and 35 μL of IP1 were added to each well of the ASE plates, and the plates were placed on a raised-bar magnetic plate until the beads had been completely captured. Next, 30 μL of each resulting supernatant was transferred to the PCR plate, and PCR was performed using 3 universal primers (2 labeled with fluorescent dyes, the third biotinylated). The reagents were denatured at 37°C for 10 min and at 95°C for 3 min, and then subjected to 34 cycles of denaturation for 35 s at 95°C, annealing for 35 s at 56°C, and extension for 2 min at 72°C, followed by a final extension for 10 min at 72°C and then incubation for 5 min at 4°C.

Binding of PCR products

Twenty microliters of each MPB reagent was added to the PCR plates, and the resulting mixtures were transferred to a filter plate. The filter plate was incubated at room temperature to allow the biotinylated DNA strand to bind to the paramagnetic particles, thereby immobilizing the double-stranded PCR products.

Preparation of the intermediate (INT) plates for BeadChip

The single-stranded fluorophore-labeled PCR products from the filter plate were washed and then eluted into an INT plate. The products from this plate were hybridized to a BeadChip; the chip was hybridized overnight in an Illumina hybridization oven, with a temperature ramp-down from 60°C to 45°C.

**Day 3.** The hybridized BeadChips were washed and then imaged using the iScan System, and the data from these images were analyzed using Illumina’s BeadStudio software.

All reagents used in the protocol were supplied by Illumina Inc., except for the Titanium Taq DNA Polymerase, which was purchased from TaKaRa Inc. (Shiga, Japan).

### Data analysis

To evaluate the validity, reliability, and reproducibility of the hearing-loss mutation-detection microarray, the results were verified through direct sequencing. Subsequently, we calculated the sensitivity and specificity, the false-positive and false-negative rates, Youden’s index (γ), and the positive likelihood rate (+LR) and negative likelihood rate (-LR) to further assess the validity. We also calculated the Kappa value, which represents reliability. Lastly, we analyzed the contribution of the examined causative mutations to the development of hearing loss. Statistical analyses were performed using SPSS 11.0 (SPSS Statistics, Inc., Chicago, IL, USA).

## Results

### Patient and audiometric characteristics

The 382 nonsyndromic SNHL patients were aged 11.78 ± 1.44 years old; 193 of the 382 patients (50.52%) were females, 189 (49.48%) were males. All 382 patients presented bilateral SNHL, 329 (86.13%) exhibited prelingual hearing impairment, and 16 (4.19%), 51 (13.35%), and 315 (82.46%) patients showed moderate, severe, and profound hearing loss, respectively. This group also included 15 patients with EVA syndrome (average age, 7.07 ± 1.45 years; 6 (40%) females, 9 (60%) males). All 15 patients with EVA syndrome presented with bilateral SNHL, 12 (80%) patients showed prelingual hearing impairment, and 3 (20%) exhibited postlingual hearing impairment. Of these 3 patients, 2 had a history of head trauma. Meanwhile, severe and profound hearing loss was present in 1 (6.67%) and 14 (93.33%) of the 15 EVA patients, respectively.

The WS patients were aged 9.04 ± 1.38 years old; 6 of these 21 patients (28.57%) were females and 15 (71.43%) were males. All 21 patients presented bilateral profound SNHL, prelingual hearing impairment, and dystopia canthorum of hair, eyes, or skin. Of these 21 individuals, 10 (47.62%) were WS1 patients and the remaining 11 (52.38%) were WS2 patients.

### Overall array performance

The call rate of the microarray was 99.375%. To evaluate the accuracy and repeatability of the hearing-loss genotyping microarray, the results were validated through Sanger sequencing. The results of this analysis demonstrated that the microarray assay was highly sensitive and specific, as few false-negative and false-positive mutations were detected. The calculated performance values were as follows (respectively): sensitivity and specificity, 98.73% and 98.34%; false-negative and false-positive rates, 1.27% and 1.66%; Youden’s index (γ), 0.97; and positive likelihood rate (+LR) and negative likelihood rate (-LR), 59.49 and 0.01. As compared with directing sequencing, the Percent Agreement was 98.44% and the Kappa value was 0.917.

### Genotype of nonsyndromic SNHL in the Chinese population

Using our microarray protocol, we detected molecular defects in 234/382 (61.26%) patients with nonsyndromic SNHL; thus, mutations were not detected in <40% of the cases (38.74%, 148/382). In summary, 48 patients were homozygous for mutations in *GJB2*, 16 for mutations in *MT-RNR1*, and 10 for mutations in *SLC26A4*; 15 patients harbored compound heterozygous mutations in *GJB2*, 14 harbored mutations in *SLC26A4*, 2 in *CDH23* (OMIM: 605516), and 1 in *MYO15A* (OMIM: 602666). Because these genes are recessive, only the SNHL patients who harbored homozygous or compound heterozygous pathological mutations were considered to present hearing impairment caused by the aforementioned mutations. Thus, through this analysis, the molecular etiology of deafness was confirmed in 23.56% (90/382) of the patients, and it involved the *GJB2*, *SLC26A4*, *CDH23*, *MYO15A*, and *MT-RNR1* genes (Tables 2 and 3). Ten patients were identified with multiple genetic mutations (Table 4), who were not confirmed molecular etiology.

**Table 2:** Summary of mutant alleles identified in patients with nonsyndromic sensorineural hearing loss (SNHL).

Gene	Nucleotide change	Amino acid change	Pathogenic type	Heterozygous	Homozygous	Mutant allele frequency	Mutant frequency
*GJB2*	c.35delG	Frameshift	R	0	1	0.26%	0.26%
	c.139G>T	p.Glu47Term	R	1	0	0.13%	0.26%
	c.235delC	Frameshift	R	33	39	14.53%	18.85%
	c.299_300delAT	Frameshift	R	14	3	2.62%	4.45%
	c.368C>A	p.Thr123Asn	R	5	0	0.65%	1.31%
	c.427C>T	p.Arg143Trp	R	0	4	1.05%	1.05%
	c.571T>C	p.Phe191Leu	R	5	0	0.65%	1.31%
	c.608T>C	p.Ile203Thr	R	32	1	4.45%	8.64%
*SLC26A4*	c.269C>T	p.Ser90Leu	R	1	0	0.13%	0.26%
	c.589G>A	p.Gly197Arg	E	2	0	0.26%	0.52%
	c.754T>C	p.Ser252Pro	R	4	0	0.52%	1.05%
	c.IVS7_2A>G	Aberrant splicing	P/E/R	34	13	7.85%	12.30%
	c.1174A>T	p.Asn392Tyr	R	3	0	0.39%	0.79%
	c.1246A>C	p.Thr416Pro	P	1	0	0.13%	0.26%
	c.IVS10_12T>A	Aberrant splicing	E/R	6	0	0.79%	1.57%
	c.1343C>A	p.Ser448Term	R	1	0	0.13%	0.26%
	c.1489G>A	p.Gly497Ser	R	2	0	0.26%	0.52%
	c.IVS14+1G>A	Aberrant splicing	P	1	0	0.13%	0.26%
	c.1975G>C	p.Val659Leu	R	4	0	0.52%	1.05%
	c.2168A>G	p.His723Arg	P/E/R	12	0	1.57%	3.14%
*CDH23*	c.2968G>A	p.Asp990Asn	R	19	0	2.49%	4.97%
	c.6604G>A	p.Asp2202Asn	R	3	0	0.39%	0.79%
	c.6823G>A	p.Arg2608His	R	25	0	3.27%	6.54%
	c.8866C>T	p.Arg2956Cys	R	5	0	0.65%	1.31%
*MYO15A*	c.3685C>T	p.Gln1229Term	R	13	0	1.70%	3.40%
	c.4351G>A	p.Asp1451Asn	R	1	0	0.13%	0.26%
	c.4669A>G	p.Lys1557Glu	R	2	0	0.26%	0.52%
	c.5189T>C	p.Leu1730Pro	R	1	0	0.13%	0.26%
	c.6337A>T	p.Ile2113Phe	R	1	0	0.13%	0.26%
	c.9478C>T	p.Leu3160Phe	R	2	0	0.26%	0.52%
*DFNB59*	c.499C>T	p.Arg167Term	R	16	0	2.09%	4.19%
*12rsRNA*	c.1555A>G	——	R	16	0	2.09%	4.19%
*PCDH15*	c.400C>G	p.Arg134Gly	R	13	0	1.70%	3.40%
	c.785G>A	p.Gly262Asp	R	1	0	0.13%	0.26%
*OTOF*	c.1273C>T	p.Arg425Term	R	1	0	0.13%	0.26%
	c.IVS28_2A>C	Aberrant splicing	R	4	0	0.52%	1.05%
	c.IVS39+1G>C	Aberrant splicing	R	2	0	0.26%	0.52%
	c.5197G>A	p.Glu1733Lys	R	2	0	0.26%	0.52%
*TRIOBP*	c.3055G>A	p.Gly1019Arg	R	7	0	0.92%	1.83%
*TMC1*	c.1334G>A	p.ArgRG445His	R	4	0	0.52%	1.05%
	c.IVS21+5G>A	Aberrant splicing	R	2	0	0.26%	0.52%
*MYO1A*	c.277C>T	p.Arg93Term	R	1	0	0.13%	0.26%
	c.2390C>T	p.Ser797Phe	R	1	0	0.13%	0.26%
	c.916G>A	p.Val306Met	R	3	0	0.39%	0.79%
*KCNQ4*	c.546C>G	p.Phe182Leu	D	4	0	0.52%	1.05%
*MYH14*	c.1126G>T	p.Gly376Cys	R	3	0	0.39%	0.79%
*MYO3A*	c.IVS8_2A>G	Aberrant splicing	R	3	0	0.39%	0.79%
*MYH9*	c.2114G>A	p.Arg705His	D	2	0	0.26%	0.52%
*TECTA*	c.249C>T	p.Thr83Met	D	2	0	0.26%	0.52%
*EYA4*	c.IVS14_12T>A	Aberrant splicing	R	2	0	0.26%	0.52%
*MYO6*	c.737A>G	p.His246Arg	R	1	0	0.13%	0.26%
*MYO7A*	c.652G>A	p.Asp218Asn	D	1	0	0.13%	0.26%
*TMPRSS3*	c.646C>T	p.Arg216Cys	R	1	0	0.13%	0.26%

D: autosomal dominant inheritance; R: autosomal recessive inheritance; P: Pendred syndrome; E: enlarged vestibular aqueduct syndrome.

**Table 3:** Genotypes of patients harboring mutations in single autosomal-recessive inherited gene.

Gene	Allele 1			Allele 2			No. of patients	Frequency
	Nucleotide change	Amino acid change	Pathogenic type	Nucleotide change	Amino acid change	Pathogenic type		
*GJB2*	c.235delC	Frameshift	R	c.235delC	Frameshift	R	39	43.33%
	c.427C>T	p.Arg143Trp	R	c.427C>T	p.Arg143Trp	R	4	4.44%
	c.608T>C	p.Ile203Thr	R	c.608T>C	p.Ile203Thr	R	1	1.11%
	c.299-300delAT	Frameshift	R	c.299-300delAT	Frameshift	R	3	3.33%
	c.35delG	Frameshift	R	c.35delG	Frameshift	R	1	1.11%
	c.235delC	Frameshift	R	c.139G>T	p.Glu47Term	R	1	1.11%
	c.235delC	Frameshift	R	c.299-300delAT	Frameshift	R	11	12.22%
	c.235delC	Frameshift	R	c.608T>C	p.Ile203Thr	R	1	1.11%
	c.235delC	Frameshift	R	c.368C>A	p.Thr123Asn	R	1	1.11%
	c.368C>A	p.Thr123Asn	R	c.608T>C	p.Ile203Thr	R	1	1.11%
*SLC26A4*	c.IVS7-2A>G	Aberrant splicing	P/E/R	c.IVS7-2A>G	Aberrant splicing	P/E/R	10	11.11%
	c.IVS7-2A>G	Aberrant splicing	P/E/R	c.2168A>G	p.His723Arg	P/E/R	5	5.56%
	c.IVS7-2A>G	Aberrant splicing	P/E/R	c.1174A>T	p.Asn392Tyr	R	2	2.22%
	c.IVS7-2A>G	Aberrant splicing	P/E/R	c.589G>A	p.Gly197Arg	E	1	1.11%
	c.IVS7-2A>G	Aberrant splicing	P/E/R	c.IVS10-12T>A	Aberrant splicing	E/R	3	3.33%
	c.2168A>G	p.His723Arg	P/E/R	c.754T>C	p.Ser252Pro	R	1	1.11%
	c.2168A>G	p.His723Arg	P/E/R	c.IVS10-12T>A	Aberrant splicing	E/R	1	1.11%
	c.1174A>T	p.Asn392Tyr	R	c.1343C>A	p.Ser448Term	R	1	1.11%
*CDH23*	c.6823G>A	p.Arg2608His	R	c.8866C>T	p.Arg2956Cys	R	1	1.11%
	c.6823G>A	p.Arg2608His	R	c.2968G>A	p.Asp990Asn	R	1	1.11%
*MYO15A*	c.3685C>T	p.Gln1229Term	R	c.9478C>T	p.Leu3160Phe	R	1	1.11%
Total							90	100.00%

D: autosomal dominant inheritance; R: autosomal recessive inheritance; P: Pendred syndrome; E: enlarged vestibular aqueduct syndrome.

**Table 4:** Genotypes of patients harboring mutations in multiple autosomal-recessive inherited genes.

Allele 1				Allele 2				No. of subjects
Gene	Nucleotide change	Amino acid change	Pathogenic type	Gene	Nucleotide change	Amino acid change	Pathogenic type	
*GJB2*	c.608T>C	p.Ile203Thr	R	*SLC26A4*	c.IVS7_2A>G	Aberrant splicing	P/E/R	1
*GJB2*	c.368C>A	p.Thr123Asn	R	*SLC26A4*	c.IVS7_2A>G	Aberrant splicing	P/E/R	1
*GJB2*	c.608T>C	p.Ile203Thr	R	*SLC26A4*	c.1975G>C	p.Val659Leu	R	1
*GJB2*	c.368C>A	p.Thr123Asn	R	*MYO3A*	c.IVS8_2A>G	Aberrant splicing	R	1
*GJB2*	c.608T>C	p.Ile203Thr	R	*MYO3A*	c.IVS8_2A>G	Aberrant splicing	R	1
*GJB2*	c.235delC	Frameshift	R	*PCDH15*	c.400C>G	p.Arg134Gly	R	1
*CDH23*	c.2968G>A	p.Asp990Asn	R	*SLC26A4*	c.IVS7_2A>G	Aberrant splicing	P/E/R	2
*CDH23*	c.2968G>A	p.Asp990Asn	R	*PCDH15*	c.400C>G	p.Arg134Gly	R	1
*CDH23*	c.6823G>A	p.Arg2608His	R	OTOF	c.IVS28_2A>C	Aberrant splicing	R	1
Total								10

The mutant frequency of *GJB2* was 36.13% (Table 2), and *GJB2* was the most prevalent causative gene among the nonsyndromic SNHL patients. In this gene, 8 types of mutations were detected, among which c.235delC was the most prevalent mutation. Furthermore, the c.235delC mutation accounted for 59.68% (111/186) of all mutant *GJB2* alleles. In addition, c.608T>C, c.299_300delAT, and c.427C>T were frequently encountered pathological mutations of *GJB2*, accounting for 18.28% (34/186), 10.20% (20/186), and 4.3% (8/186) of all mutations in this allele, respectively.

*SLC26A4* was the second most prevalent causative gene among the nonsyndromic SNHL patients examined (20.68%; Tables 2 and 3). Eleven distinct mutations were detected in *SLC26A4*, among which c.IVS7-2A>G was the most prevalent mutation, accounting for 58.43% (52/89) of all mutant *SLC26A4* alleles. Meanwhile, the c.2168A>G, c.IVS10-12T>A, c.754T>C, and c.1975G>C mutations accounted for 13.48% (12/89), 6.74% (6/89), 4.49% (4/89), and 4.49% (4/89) of the mutant *SLC26A4* alleles, respectively.

Furthermore, the results obtained using our protocol revealed that the incidence of genetic dominant defects in the patient group tested was 2.88% (11/382). The molecular etiology was confirmed for each of these 11 patients, and it involved the *KCNQ4* (OMIM: 603537), *WFS1* (OMIM: 606201), *TECTA* (OMIM: 602574), or *MYH9* (OMIM: 160775) genes (Table 5). Because these 4 genes showed a dominant inheritance pattern, we confirmed the sequence variants of the probands by DNA sequencing, and verified the co-segregation in their families. The results identified the following mutations harbored by the patients: *KCNQ4* c.546C>G, *WFS1* c.1846G>T, *TECTA* c.249C>T, and *MYH9* c.2114G>A (Table 4).

**Table 5:** Genotypes of patients harboring mutations in autosomal-dominant inherited genes.

Gene	Allele 1			Allele 2			No. of patients	Frequency
	Nucleotide change	Amino acid change	Pathogenic type	Nucleotide change	Amino acid change	Pathogenic type		
*KCNQ4*	c.546C>G	p.Phe182Leu	D	——	——	——	4	36.36%
*WFS1*	c.1846G>T	p.Ala616Ser	D	——	——	——	3	27.27%
*TECTA*	c.249C>T	p.Thr83Met	D	——	——	——	2	18.18%
*MYH9*	c.2114G>A	p.Arg705His	D	——	——	——	2	18.18%
Total							11	100.00%

D: autosomal dominant inheritance.

Heterozygous mutations were also confirmed in the following 11 nuclear recessive genes: *DFNB59* (OMIM: 610219), *PCDH15* (OMIM: 605514), *OTOF* (OMIM: 603681), *TRIOBP* (OMIM: 609761), *TMC1* (OMIM: 606706), *MYO1A* (OMIM: 601478), *MYH14* (OMIM: 608568), *MYO3A* (OMIM: 606808), *EYA4* (OMIM: 601316), *MYO6* (OMIM: 600970), and *TMPRSS3* (OMIM: 605511). As shown in Table 2, 4.19% (16/382), 3.66% (14/382), and 2.45% (9/382) of the patients were heterozygous for mutant *DFNB59*, *PCDH15*, and *OTOF* alleles, respectively. Last, no changes were identified in any of the patients in the following genes: *COCH* (OMIM: 603196), *COL11A2* (OMIM: 120290), *CRYM* (OMIM: 123740), *DFNA5* (OMIM: 608798), *DIAPH1* (OMIM: 602121), *EDN3* (OMIM: 613265), *EDNRB* (OMIM: 277580), *FOXI1* (OMIM: 274600), *GJB3* (OMIM: 603324), *GJB6* (OMIM: 604418), *KIAA1199* (OMIM: 608366), *LHFPL5* (OMIM: 609427), *LRTOMT* (OMIM: 612414), *MARVELD2* (OMIM: 610572), *OTOA* (OMIM: 607038), *PAX3* (OMIM: 606597), *POU4F3* (OMIM: 602460), *RDX* (OMIM: 179410), *SLC26A5* (OMIM: 604943), *SOX10* (OMIM: 602229), *TMIE* (OMIM: 607237), and *WHRN* (*DFNB31*; OMIM: 607928).

Thus, the molecular etiology was confirmed in 30.63% (117/382) of the children with nonsyndromic SNHL, and 76.92% (90/117) of these probands harbored pathogenic recessive mutations, 9.4% (11/117) carried pathogenic dominant mutations, and 13.68% (16/117) harbored pathogenic mutations in the mitochondrial 12S rRNA coding sequence.

### Genotypes of Chinese EVA patients

The nonsyndromic SNHL patients examined in this study included 15 patients with EVA syndrome, and molecular defects in *SLC26A4* were detected in 11 of these patients (73.33%) using our protocol. The results presented in Table 6 show that among these 11 individuals, 2 (18.18%) were homozygous for the c.IVS7-2A>G mutation, 5 (45.45%) were compound heterozygous for 2 pathogenic mutations, 1 (9.09%) was heterozygous for c.1489G>A, 1 (9.09%) was heterozygous for c.2168A>G, and 2 (18.18%) were heterozygous for c.IVS7-2A>G. Thus, 7 of the 15 patients (46.67%) patients were confirmed to be homozygous or compound heterozygous for biallelic mutations in *SLC26A4*. The allele frequencies of IVS7-2A>G and 2168A>G were 30% (9/30) and 10% (3/30), respectively, in the EVA patient group.

**Table 6:** Genotypes of the enlarged vestibular aqueduct (EVA) patients harboring mutations in *SLC26A4*.

No.	Allele 1			Allele 2			No. of patients
	Nucleotide change	Amino acid change	Pathogenic type	Nucleotide change	Amino acid change	Pathogenic type	
1	c.IVS7-2A>G	Aberrant splicing	P/E/R	c.IVS7-2A>G	Aberrant splicing	P/E/R	2
2	c.IVS7-2A>G	Aberrant splicing	P/E/R	c.2168A>G	p.His723Arg	P/E/R	1
3	c.1174A>T	p.Asn392Tyr	R	c.1343C>A	p.Ser448Term	R	1
4	c.IVS7-2A>G	Aberrant splicing	P/E/R	c.IVS10-12T>A	Aberrant splicing	E/R	2
5	c.IVS10-12T>A	Aberrant splicing	E/R	c.2168A>G	p.His723Arg	P/E/R	1
6	c.1489G>A	p.Gly497Ser	R	——	——	——	1
7	c.2168A>G	p.His723Arg	P/E/R	——	——	——	1
8	c.IVS7-2A>G	Aberrant splicing	P/E/R	——	——	——	2

R: autosomal recessive inheritance; P: Pendred syndrome; E: EVA.

### Genotypes of Chinese WS patients

We determined that 4 out of 15 patients (26.67%) carried mutations in genes related to WS: 1 patient was heterozygous for *EDN3* c.293C>A, 1 was heterozygous for *PAX3* c.238C>G, 1 was heterozygous for *PAX3* c.808C>T, and 1 carried only one *MITF* c.651G>T mutation. The clinical features and genotypes of these 4 WS patients are listed in Table 7.

**Table 7:** Clinical features and genotypes of Waardenburg syndrome (WS) patients carrying identified mutations.

No.	Type	Onset of deafness	Sex	Level of hearing loss	Pigmentary disturbance	Dystopia canthorum	Aganglionic megacolon	Family history	Gene	Nucleotide change	Amino acid change
1	WS1	Birth	F	Profound	+	+	−	No	*EDN3*	Heterozygous c.293C>A	p.Thr98Lys
2	WS1	Birth	M	Profound	+	+	−	No	*PAX3*	Heterozygous c.238C>G	p.His80Asp
3	WS1	Birth	F	Profound	+	−	−	Yes	*PAX3*	Heterozygous c.808C>T	p.Arg270Cys
4	WS2	Birth	M	Profound	+	−	−	Yes	*MITF*	Heterozygous c.650G>T	p.Arg217Ile

### Genotypes of the control group

*GJB2* c.235delC was the only mutation detected in the control group, being present in 1 of the 60 control participants with normal hearing (mutation carrier frequency: 1.67%). The other mutations were not detected in the control group.

## Discussion

Deafness, the most common sensory-nerve disease worldwide [1], not only causes hearing disability, but also affects mental health, and concurrently places a heavy burden on the patient’s family and the society. Although the use of hearing aids, cochlear implants, and other methods can improve or restore hearing function in some patients, these approaches do not explain the cause of deafness or provide methods to prevent the occurrence of the same phenotype in the patients’ children. Approximately half of all congenital deafness cases are associated with genetic factors, and genetic screening and diagnosis both simplify the entire progress of diagnosis, therapy, and prognosis, and help to explain the etiology and predict the possibility of inheritance [11]. However, the approach required for obtaining a genetic diagnosis of hereditary deafness is challenging for the following reasons: first, there are currently no optimal strategies to obtain such diagnoses. Because of the high genetic heterogeneity of genetic hearing loss, no clear and confirmed relationship exists between each type of hearing loss and the identified genetic variants. Indeed, patients with identical same clinical manifestations might present distinct genotypes, or vice versa. Although certain strategies for detecting variants have been previously suggested [1, 12], these are not suitable for people of all ethnic groups living in distinct geographical regions. Second, there are currently no optimal technologies available for diagnosis of hereditary deafness. Sanger sequencing, which has been the screening method used for genetic research and clinical genetic diagnostics for almost 40 years, is the most definitive method [13]. However, using Sanger sequencing to screen each disease-associated gene to identify causative variants is time-consuming and expensive. Thus, gene chips have typically been used in the screening and diagnosis of hearing loss. The advantages of gene-chip technology are its low cost, simplicity, and availability; however, the technology can only be used to detect specific known mutations [6]. Another technology that has been developed is next-generation sequencing (NGS; also called second-generation sequencing), which offers high throughput and high sequencing depth, and is considered as a suitable method for molecular diagnosis of hearing loss; however, the increased output of NGS is associated with an increased error rate, which varies from 0.1% to 2% [14]. Moreover, analysis of the data obtained via this technique remains a bottleneck encountered by geneticists [15]. Given these drawbacks, we sought to develop a tool that could be used as a first-pass screen prior to NGS-based screening.

We developed a hearing-loss-detection microarray featuring 240 selected mutations in 1 mitochondrial gene and 46 nuclear genes. The variants in this microarray represent the genes that are currently those most frequently involved in deafness, most of which have been reported more than twice previously in the Chinese population. As such, ours is the first microarray to cover both nonsyndromic and syndromic SNHL. In Table 8, we compare our microarray with a few previously reported technologies. No currently available technology for molecular testing of SNHL in China is as efficient or comprehensive as the hearing-loss microarray presented here. Our microarray is easy to operate, and is highly advantageous because of its versatility. The call rate of the microarray was 99.375%. Moreover, the assay performed in this study was highly sensitive and specific, as indicated by the low detection rates of false-negative and false-positive mutations. Specifically, the sensitivity and specificity were 98.73% and 98.34%, respectively. Furthermore, for a given test, a +LR of >10 and -LR of <0.10 theoretically indicate a high validity for the test in clinical application. In our study, +LR and -LR were 59.49 and 0.01, respectively. Meanwhile, Youden’s index serves as an index for summarizing the performance of a diagnostic test, and its value ranges from 0 to 1, with 0 indicating that a test is unreliable and 1 indicating that the test is optimal. In our study, Youden’s index was 0.97, demonstrating that the test is highly reliable. Kappa is a statistical measure of the agreement in ratings between two tests, and its value ranges from -1 to 1, with 1 indicating complete consistency between two tests. When comparing the microarray results with those obtained by Sanger sequencing, the Kappa value for our microchip was 0.917, which further demonstrated the reliability of our microarray.

**Table 8:** Comparison of several methods for the genetic detection of hearing loss.

	DNA sequencing technology	Screened mutations/genes	Advantages	Disadvantages	Reference
**Invader assay**	Invader assay	41 mutations/9 genes	High accuracy	Not comprehensive	[16]
**OtoChip**	Resequencing microarray	13 deafness genes	Efficiency, low cost	Expensive and not comprehensive	[17]
**HHL APEX**	Single base pair primer extension	198 mutations/8 genes	Efficiency, flexibility	Not comprehensive	[18]
**MQ-LCR**	Multiplex quantitative ligase chain reaction	5 mutations/3 genes	Low cost, convenience	Not comprehensive	[19]
**CapitalBio**	Arrayed primer extension	9 mutations/4 genes	Low cost, convenience	Not comprehensive	[7]
**SNaPshot**	Minisequencing technique	7 mutations/3 genes	Efficiency, low cost	Not comprehensive	[20]
GoldenGate*	Single base pair primer extension	240 mutations/46 genes	Comprehensive, flexible, accurate	Long indels cannot be included	

*The microarray developed in this study.

Among the variants in all identified causative autosomal-recessive genes, *GJB2* variants represent the most common known cause of autosomal-recessive SNHL in several ethnicities, and *GJB2* is the currently the only gene examined in most diagnostic laboratories [13]. The occurrence ratio of *GJB2* mutations in the general population is around 1/33 [21]. *GJB2* is a common pathogenic gene and its variants have been reported in, for example, Europe, Tunisia, Lebanon, Australia, and New Zealand. In China, 21%–27% of prelingual deafness is due to *GJB2* mutations, the most common of which is 235delC [22]. In this study, 36.13% of our patients presented *GJB2* mutations together with nonsyndromic SNHL, and more than half (53.85%, 63/117) of the nonsyndromic SNHL patients with an identified molecular etiology were diagnosed with homozygous or compound heterozygous mutations in *GJB2*. *GJB2* c.235delC was the most common mutation identified, accounting for 59.68% of the mutant *GJB2* alleles, which is comparable to the results of earlier studies [1]. Three other key causative mutations identified here were *GJB2* c.427C>T, c.608T>C and c.299_300del. To clarify the genetic etiology for the patients who only carry one mutation, the entire coding sequence of *GJB2* must be analyzed for the possible existence of a second mutant allele.

As the major genetic contributor to nonsyndromic SNHL, PS, and EVA, *SLC26A4* might be the second most frequent causative gene of hereditary hearing loss worldwide. In this study, 20.51% (24/117) of the nonsyndromic SNHL patients with an identified molecular etiology were diagnosed with homozygous or compound heterozygous mutations in *SLC26A4*. c.IVS7-2A>G was the most prevalent *SLC26A4* mutation, accounting for 58.43% (52/89) of all mutations in this gene. *SLC26A4* c.2168A>G, c.754T>C, and c.1975G>C were next most prevalent pathological mutations. Among the EVA patients, 46.67% (7/15) carried causal mutations in *SLC26A4*, and c.IVS7-2A>G accounted for 31.58% (6/19) of all mutant *SLC26A4* alleles.

The mitochondrial 12S rRNA 1555A>G mutation is another hot spot associated with both aminoglycoside-induced hearing loss and SNHL in China. Indeed, the 1555A>G mutation was reported to account for up to 7.5% of the deaf in the Chinese population [23–25]. In our study, 4.08% (16/382) of the patients with nonsyndromic SNHL were homoplastic for 1555A>G, and each of these patients exhibited profound and prelingual SNHL. Moreover, for 2 patients with prelingual and profound SNHL, the molecular etiology was identified to involve *CDH23*, and these patients carried the compound heterozygous mutations c.6823G>A and c.8866C>T, and c.6823G>A and c.2968G>A, respectively. In addition, another patient with prelingual and profound SNHL was found to harbor the compound heterozygous mutations *MYO15A* c.3685C>T and c.9478C>T. Notably, these 3 types of compound causative mutations have not been previously reported in the Chinese population. Altogether, 11 patients were confirmed to harbor dominant mutations in 4 genes: *KCNQ4*, *WFS1*, *TECTA*, and *MYH9*. We confirmed the sequence variants of these probands by Sanger sequencing analysis, and verified the co-segregation in their families. Furthermore, mutation carriers were confirmed in the case of 15 autosomal genes, including *GJB2*, *SLC26A4*, *CDH23*, *MYO15A*, *DFNB59*, *PCDH15*, *OTOF*, *TRIOBP*, *TMC1*, *MYO1A*, *MYH14*, and *MYO3A*. In particular, the carrier frequencies for *CDH23*, *MYO15A*, *DFNB59*, *PCDH15*, and *OTOF* were 14.17%, 5.45%, 4.36%, 3.81%, and 2.45%, respectively. For patients who were heterozygous for only a single mutation (Table 2) or multiple mutations of different genes (Table 4), these mutations were highly suggestive of a genetic cause and the patients’ samples required further sequencing analyses. Increased attention should be paid to these genes when screening for nonsyndromic SNHL in China.

Genotype-to-phenotype correlation is also important, because different mutations in the same gene can lead to different phenotypes[12]. Most nonsyndromic autosomal forms cause prelingual hearing loss which is severe to profound[26]. In our study, 63 nonsyndromic SNHL patients with an identified molecular etiology were diagnosed with homozygous or compound heterozygous mutations in *GJB2*. All 63 of these patients presented prelingual SNHL and most of the patients (96.83%, 61/63) showed profound SNHL, and other 2 patients were severe hearing loss. Among 24 patients identified with homozygous or compound heterozygous mutations in *SLC26A4*, 21 (87.5%, 21/24) presented prelingual SNHL and 23 (95.83%, 23/24) presented profound SNHL, 1 presented severe SNHL. Furthermore, each of the patients who were diagnosed with homozygous or compound heterozygous mutations in mitochondrial 12S rRNA, *CDH23*, *MYO15A*, *WFS1*, *KCNQ4*, *TECTA*, and *MYH9* exhibited profound and prelingual SNHL. Most of these patients were consistent with genotype—phenotype relationship as previous reports[11, 26–28]. However, two patients who respectively carried *KCNQ* c.546C>G and *MYH9* c.2114G>A presented prelingual and profound hearing impairment. This is not consistent with the progressive hearing loss of *KCNQ* and *MYH9* mutants as reported[11, 29].

WS is the most common autosomal-dominant syndromic deafness. While the incidence rate of WS is 1/212,000 [30], the genuine incidence rate is suspected to be 1/42,000 because the incomplete penetrance rate is 20% [31], which accounts for 2%–5% of congenital deafness [32] and 0.9%–2.8% of deaf-mutism [33]. The major clinical features of WS are sensorineural deafness and abnormal pigment distribution on the skin, hair, iris, and inner ear. On the basis of these phenotypes, WS can be divided into 4 types (WS1–WS4) [34], with WS1 and WS2 being the most prevalent. To date, 6 genes have been confirmed to be related to WS: *MITF* (OMIM: 156845), *PAX3* (OMIM: 606597), *SOX10* (OMIM: 602229), *SNAI2* (602150), *ENDRB* (OMIM: 277580), and *EDN3* (OMIM: 613265) [35]. In our study, we selected 14 mutations from 5 genes, most of which have been reported in Chinese WS patients. Twenty-one WS patients were evaluated using our microarray, and the molecular etiology was confirmed for 4 (19.05%) of these 21 patients. Of these, 3 were WS1 patients and 1 was a WS2 patient, and all 4 presented congenital and profound SNHL (Table 6). We suggest that when screening for WS patients in China, increased attention must be devoted to the following 4 previously reported causal mutations: *EDN3* c.293C>A [35], *PAX3* c.238C>G [36], *PAX3* c.808C>T [36–39], and *MITF* c.651G>T [36].

In summary, genetic defects were detected in up to 61.26% (234/382) of the nonsyndromic SNHL patients in our study, and the inherited molecular etiology was confirmed in 30.63% (117/382) of these patients. Our results show that, among the patients tested, 76.92% (90/117) of hereditary nonsyndromic deafness was autosomal recessive, 9.4% (11/117) was autosomal dominant, and 13.68% (16/117) was associated with a maternally inherited mitochondrial gene. This study also supports the notion that the mutations in *GJB2*, *SLC26A4*, and the mitochondrial gene are the major genetic causes of nonsyndromic SNHL in China, and account for approximately 53.85% (63/117), 20.51% (24/117), and 13.68% (16/117) of these cases, respectively. Genetic defects were identified in 19.05% of the WS patients, and genetic causative mutations were confirmed in 46.67% of the EVA patients. Thus, the array presented here comprises an optimal platform for screening and detecting hereditary hearing loss, and the use of this array could yield valuable findings. The mutation screening could help the deaf in avoiding marriage between people carrying the same mutations. Moreover, the platform can be used for neonatal screening. Once the etiology of the hearing loss has been identified, early interventions, such as the use of hearing aids, cochlear implants, medicines, and life guides, can contribute substantially toward improving the quality of life in people with hearing impairment [40].

## Conclusions

We successfully developed an accurate and comprehensive genetic tool for SNHL diagnosis, and were able to use this tool to detect genetic defects associated with nonsyndromic SNHL and causative mutations of WS in up to 61.26% and 19.05% of the Chinese SNHL and WS patients tested, respectively. These attributes make the method presented here highly useful. Indeed, our method could be used for first-pass screening before NGS screening. However, no data on PS and US patients were analyzed in this study, as these 2 syndromes were not diagnosed in any patient in our department. Thus, for future versions, we will replace some of the featured mutations and optimize the microarray based on large-scale screening.

## Supporting Information

S1 Table The 240 variants detectable with the microarray. (DOC)
