# Supplementary material for: Application of a New Genetic Deafness Microarray for Detecting Mutations in the Deaf in China
Source: PLoS One. 2016 Mar 28;11(3):e0151909. doi: 10.1371/journal.pone.0151909 (PMC4809548; doi:10.1371/journal.pone.0151909)
Supplement: S1 Table — (DOC) [file pone.0151909.s001.doc]

**S1 Table. 240 Variants Detectable w**ith the microarray

| **Genes**  **(cDNA sequence and OMIM number)** | **Location** | **Amino Acid Change** | **Nucleotide Change** | **Phenotype** |
| --- | --- | --- | --- | --- |
| *CDH23* (NM_022124.3) (OMIM: 605516) | 10q21-q22 | Asp124Gly | A>G | NS |
|  |  | Pro240Leu | C>T | NS |
|  |  | Arg301Gln | G>A | NS |
|  |  | Asn452Ser | A>G | NS |
|  |  | Leu480Gln | T>A | NS |
|  |  | Arg582Gln | G>A | NS |
|  |  | Asp990Asn | G>A | NS |
|  |  | Gly1186Asp | G>A | NS |
|  |  | Asp1341Asn | G>A | NS |
|  |  | Asp2202Asn | G>A | NS |
|  |  | Arg2465Trp | C>T | NS |
|  |  | Arg2608His | G>A | NS |
|  |  | Pro3059Thr | C>A | NS |
|  |  | Arg3189Trp | C>T | NS |
|  |  | Arg2956Cys | C>T | NS |
|  |  | Gln1716Pro | A>C | NS |
|  |  | Arg2029Trp | C>T | NS |
| *COCH* (NM_004086.1) (OMIM: 603196) | 14q12-q13 | Pro51Ser | C>T | NS |
|  |  | Gly88Glu | G>A | NS |
|  |  | Ile109Thr | T>C | NS |
|  |  | Met512Thr | T>C | NS |
|  |  | Cys542Tyr | G>A | NS |
| *COL11A2* (NM_080680.2) (OMIM: 120290) | 6p21.3 | Gly808Glu | G>A | NS |
|  |  | Pro621Thr | C>A | NS |
| *CRYM* (NM_001888.2) (OMIM: 123740) | 16p13.11-p12.3 | Lys314Thr | A>C | NS |
| *DFNA5* (NM_004403.2) (OMIM: 608798) | 7p15 | IVS7-6 | C>G | NS |
|  |  | IVS8+4 | A>G | NS |
| *DIAPH1* (NM_005219.4) (OMIM: 602121) | 5q31 | IVS17+1 | G>T | NS |
| *EDN3* (NM_207034.1) (OMIM: 613265) | 20q13.2-q13.3 | Cys159Phe | G>T | WS |
|  |  | Thr98Lys | C>A | WS |
| *EDNRB* (NM_004452.2) (OMIM: 277580) | 14q24.3 | Arg201Term | C>T | WS |
|  |  | Gly170Glu | G>A | WS |
|  |  | Ala110Val | C>T | NS |
|  |  | Val342Leu | G>T | NS |
| *EYA4* (NM_004100.3) (OMIM: 601316) | 6q23 | IVS14-12 | T>A | NS |
|  |  | Arg587Term | C>T | NS |
| *FOXI1* (NM_012188.4) (OMIM: 274600) | 5q34 | Arg267Gln | G>A | PS |
| *GJB2* (NM_004004.4) (OMIM: 121011) | 13q11-q12 | Frameshift | 35delG | NS |
|  |  | Glu47Term | G>T | NS |
|  |  | Frameshift | 235del C | NS |
|  |  | Frameshift | 299_300del AT | NS |
|  |  | Thr123Asn | C>A | NS |
|  |  | Arg143Trp | C>T | NS |
|  |  | Met163Val | A>G | NS |
|  |  | Phe191Leu | T>C | NS |
|  |  | Ile203Thr | T>C | NS |
| *GJB3* (NM_024009.2) (OMIM: 603324) | 1p34 | Arg180Term | C>T | NS |
|  |  | Ile141Val | A>G | NS |
| *GJB6* (NM_006783.2) (OMIM: 604418) | 13q12 | Ala40Val | C>T | NS |
| *KCNQ4* (NM_004700.2) (OMIM: 603537) | 1p34 | Phe182Leu | C>G | NS |
|  |  | Trp242Term | G>A | NS |
|  |  | Asp262Val | A>T | NS |
|  |  | Gly285Cys | G>T | NS |
|  |  | Gly321Ser | G>A | NS |
|  |  | Thr501Thr | C>T | NS |
| *KIAA1199* (NM_018689.1) (OMIM: 608366) | 15q24 | Arg187Cys | C>T | NS |
|  |  | His783Tyr | C>T | NS |
| *LHFPL5* (NM_182548.3) (OMIM: 609427) | 6p21.31 | Tyr127Cys | A>G | NS |
| *LRTOMT* (NM_001145308) (OMIM: 612414) | 11q13.4 | Arg81Gln | G>A | NS |
|  |  | Glu110Lys | G>A | NS |
| *MARVELD2* (NM_001038603.1) (OMIM: 610572) | 5q13.2 | Arg500Term | C>T | NS |
|  |  | IVS3-1 | G>A | NS |
|  |  | IVS4+2 | T>C | NS |
| *MITF* (NM_000248.3) (OMIM: 156845) | 3p14.2-p14.1 | Phe7Cys | A>G | WS |
|  |  | Ala111Val | C>T | WS |
|  |  | Arg217Ile | G>T | WS |
|  |  | Arg255Term | C>T | WS |
| *MYH14* (NM_024729.3) (OMIM: 608568) | 19q13.33 | Gly376Cys | G>T | NS |
|  |  | Leu976Phe | C>T | NS |
| *MYH9* (NM_002473.3) (OMIM: 160775) | 22q13.1 | Asp1447Val | A>T | NS |
|  |  | Arg705His | G>A | NS |
| *MYO15A* (NM_016239.3) (OMIM: 602666) | 17p11.2 | Gln1229Term | C>T | NS |
|  |  | IVS4+1 | G>T | NS |
|  |  | Tyr1392Term | C>A | NS |
|  |  | Asp1451Asn | G>A | NS |
|  |  | Lys1557Glu | A>G | NS |
|  |  | Cys1666Term | C>A | NS |
|  |  | Leu1730Pro | T>C | NS |
|  |  | Gly1831Val | G>T | NS |
|  |  | Ile2113Phe | A>T | NS |
|  |  | Gly2244Glu | G>A | NS |
|  |  | Gln2716His | G>T | NS |
|  |  | Asp2720His | G>C | NS |
|  |  | IVS50_1 | G>C | NS |
|  |  | IVS54+1 | G>A | NS |
|  |  | Leu3160Phe | C>T | NS |
| *MYO1A* (NM_005379.2) (OMIM: 601478) | 12q13-q14 | Gly674Asp | G>A | NS |
|  |  | Arg93Term | C>T | NS |
|  |  | Glu385Asp | G>T | NS |
|  |  | Ser797Phe | C>T | NS |
|  |  | Ser910Pro | T>C | NS |
|  |  | Val306Met | G>A | NS |
| *MYO3A* (NM_017433.4) (OMIM: 606808) | 10p11.1 | IVS8_2 | A>G | NS |
|  |  | IVS17_12 | G>A | NS |
|  |  | Tyr1042Term | T>G | NS |
| *MYO6* (NM_004999.3) (OMIM: 600970) | 6q13 | IVS23+2428 | T>G | NS |
|  |  | Arg1166Term | C>T | NS |
|  |  | Arg849Term | C>T | NS |
|  |  | Cys442Tyr | G>A | NS |
|  |  | Glu216Val | A>T | NS |
|  |  | His246Arg | A>G | NS |
| *MYO7A* (NM_000260.3) (OMIM: 276903) | 11q13.5 | IVS3_2 | A>G | NS |
|  |  | Asp218Asn | G>A | NS |
|  |  | Arg244Pro | G>C | NS |
|  |  | Met599Ile | G>A | NS |
|  |  | Gly671Ser | G>A | NS |
|  |  | Gly722Arg | G>C | NS |
|  |  | IVS19-1 | G>T | US |
|  |  | Arg1240Gln | G>A | US |
|  |  | Ser1666Cys | A>T | US |
|  |  | Tyr1719Cys | A>G | US |
|  |  | Leu1954Ile | C>A | US |
| *OTOA* (NM_144672.3) (OMIM: 607038) | 16p12.2 | IVS12+2 | t>c | NS |
| *OTOF* (NM_194248.1) (OMIM: 603681) | 2p23.1 | IVS5+1 | A>G | NS |
|  |  | Arg237Term | C>T | NS |
|  |  | Gln255His | G>C | NS |
|  |  | Arg425Term | C>T | NS |
|  |  | Pro490Gln | C>A | NS |
|  |  | Ile515Thr | T>C | NS |
|  |  | Trp536Term | G>A | NS |
|  |  | Leu573Arg | T>G | NS |
|  |  | Arg708Term | C>T | NS |
|  |  | Glu747Term | G>T | NS |
|  |  | Arg794His | G>A | NS |
|  |  | Arg822Trp | C>T | NS |
|  |  | Cys883Term | C>A | NS |
|  |  | IVS24+1 | G>A | NS |
|  |  | Leu1011Pro | T>C | NS |
|  |  | Ala1090Glu | C>A | NS |
|  |  | Arg1134Term | C>T | NS |
|  |  | Leu1138Pro | T>C | NS |
|  |  | IVS28_2 | A>C | NS |
|  |  | Trp1425Term | G>A | NS |
|  |  | Gly1451Term | G>T | NS |
|  |  | Arg1495Term | C>T | NS |
|  |  | Arg1520Gln | G>A | NS |
|  |  | IVS39+1 | G>C | NS |
|  |  | Glu1733Lys | G>A | NS |
|  |  | Phe1795Cys | T>G | NS |
|  |  | Pro1825Ala | C>G | NS |
|  |  | Arg1939Gln | G>A | NS |
|  |  | Pro1987Arg | C>G | NS |
| *PAX3* (NM_181457.3) (OMIM: 606597) | 2q35 | His80Asp | C>G | WS |
|  |  | Arg223Term | C>T | WS |
|  |  | Arg270Cys | C>T | WS |
|  |  | Tyr312Term | C>A | WS |
| *PCDH15* (NM_033056.3) (OMIM: 605514) | 10q21.1 | Arg134Gly | C>G | NS |
|  |  | Gly262Asp | G>A | NS |
| *DFNB59* (NM_001042702.3) (OMIM: 610219) | 2q31.2 | Arg167Term | C>T | NS |
|  |  | Thr54Ile | C>T | NS |
| *POU3F4* (NM_000307.2) (OMIM: 300039) | Xq21.1 | Lys202Term | A>T | NS |
|  |  | Ser228Leu | C>T | NS |
|  |  | Pro303Ser | C>T | NS |
|  |  | Arg323Gly | C>G | NS |
| *POU4F3* (NM_002700.1) (OMIM: 602460) | 5q31 | Leu289Phe | C>T | NS |
| *RDX* (NM_002906.3) (OMIM: 179410) | 11q23 | Asp578Asn | G>A | NS |
|  |  | Gln155Term | C>T | NS |
| *SLC26A4* (NM_000441.1) (OMIM: 605646) | 7q31 | Ser57Term | C>A | NS |
|  |  | Ser90Leu | C>T | NS |
|  |  | Pro112Ser | C>T | EVA |
|  |  | Met147Thr | T>C | EVA |
|  |  | Gly197Arg | G>A | EVA |
|  |  | Ser252Pro | T>C | NS |
|  |  | Met283Ile | G>C | EVA |
|  |  | IVS7-2 | A>G | PS/ EVA/ NS |
|  |  | IVS8+1 | G>A | PS |
|  |  | Phe335Leu | T>C | PS |
|  |  | Ala372Val | C>T | NS |
|  |  | Asn392Tyr | A>T | NS |
|  |  | Thr416Pro | A>C | PS |
|  |  | IVS10-12 | T>A | EVA/ NS |
|  |  | Leu445Trp | T>G | PS |
|  |  | Ser448Term | C>A | NS |
|  |  | Arg470His | G>A | NS |
|  |  | Gly497Ser | G>A | NS |
|  |  | IVS14+1 | G>A | PS |
|  |  | IVS14-1 | G>A | EVA |
|  |  | Tyr556Cys | A>G | PS |
|  |  | Leu597Ser | T>C | PS |
|  |  | Val659Leu | G>C | NS |
|  |  | His723Arg | A>G | PS/ EVA/ NS |
|  |  | Arg776Cys | C>T | EVA |
| *SLC26A5* (NM_198999.1) (OMIM: 604943) | 7q22.1 | Arg150Gln | G>A | NS |
| *SOX10* (NM_006941.3) (OMIM: 602229) | 22q13.1 | IVS4-2 | A>T | WS |
|  |  | Term467Lys | T>A | WS |
| *TECTA* (NM_005422.2) (OMIM: 602574) | 11q22-q24 | Thr83Met | C>T | NS |
|  |  | Asn864Lys | C>A | NS |
|  |  | Cys1057Ser | T>A | NS |
|  |  | Cys1352Tyr | G>A | NS |
|  |  | Cys1619Ser | G>C | NS |
|  |  | Leu1820Phe | C>T | NS |
|  |  | Tyr1870Cys | A>G | NS |
|  |  | Arg1890Cys | C>T | NS |
|  |  | Arg2021His | G>A | NS |
|  |  | IVS9+1 | G>A | NS |
| *TMC1* (NM_138691.2) (OMIM: 606706) | 9q21.12 | IVS10-8 | T>A | NS |
|  |  | Pro274Leu | C>T | NS |
|  |  | IVS13+1 | G>A | NS |
|  |  | Val372Met | G>A | NS |
|  |  | Arg445His | G>A | NS |
|  |  | Arg512Term | C>T | NS |
|  |  | Asp572His | G>C | NS |
|  |  | Trp588Term | G>A | NS |
|  |  | Met654Val | A>G | NS |
|  |  | Ser668Arg | T>A | NS |
|  |  | IVS21+5 | G>A | NS |
| *TMIE* (NM_147196.1) (OMIM: 607237) | 3p21 | Arg81Cys | C>T | NS |
| *TMPRSS3* (NM_024022.1) (OMIM: 605511) | 21q22.3 | Ala90Thr | G>A | NS |
|  |  | Ala138Glu | C>T | NS |
|  |  | Cys194Phe | G>T | NS |
|  |  | Arg216Cys | C>T | NS |
|  |  | Trp251Cys | G>C | NS |
|  |  | Ala306Thr | G>A | NS |
|  |  | PRO404LEU | C>T | NS |
|  |  | Cys425Arg | T>C | NS |
| *TRIOBP* (NM_001039141.1) (OMIM: 609761) | 22q13.1 | Gly1019Arg | G>A | NS |
|  |  | Arg1117Term | C>T | NS |
|  |  | Arg347Term | C>T | NS |
| *USH2A* (NM_206933.1) (OMIM: 608400) | 1q41 | Ala125Thr | G>A | US |
|  |  | Cys419Phe | G>T | US |
|  |  | Arg626Term | C>T | US |
|  |  | Cys759Phe | G>T | US |
|  |  | Cys934Trp | T>G | US |
|  |  | Trp1263Term | G>T | US |
|  |  | Gly1734Arg | G>C | US |
|  |  | IVS32+1 | G>A | US |
|  |  | Ivs42_2 | A>G | US |
|  |  | Trp2744Cys | G>C | US |
|  |  | Glu3411Ala | A>C | US |
|  |  | Thr3936Pro | A>C | US |
|  |  | Tyr4801Term | C>G | US |
| *WFS1* (NM_006005.2) (OMIM: 606201) | 4p16 | Arg161Gln | G>A | NS |
|  |  | Lys193Gln | A>C | NS |
|  |  | Ala616Ser | G>T | NS |
|  |  | Tyr669His | T>C | NS |
|  |  | Ala716Thr | C>T | NS |
|  |  | Val779Met | G>A | NS |
|  |  | Arg859Pro | G>C | NS |
| *WHRN (DFNB31)* (NM_015404.3) (OMIM: 607928) | 9q32-q34 | Gln103Term | C>T | NS |
| 12rsRNA | Mitochondra | 12rsRNA 1555 | A>G | NS |

**NOTE:** NS represents nonsyndromic SNHL; US represents Usher syndrome; WS represents Waardenburg syndrome; PS represents Pendred syndrome; EVA represents enlarged vestibular aqueduct.
